# High-dose use of pregabalin and gabapentin in France: a retrospective, population-based cohort study

**DOI:** 10.1016/j.lanepe.2025.101424

**Published:** 2025-08-07

**Authors:** Thomas Soeiro, Marina Uras, Émilie Jouanjus, Maryse Lapeyre-Mestre, Joëlle Micallef

**Affiliations:** a UMR 1106, INS, Inserm, Aix-Marseille University, Marseille, France; b Department of Clinical Pharmacology and Drug Surveillance, Marseille University Hospital, Marseille, France; c Department of Medical and Clinical Pharmacology, Toulouse University Hospital, Toulouse, France; d UMR 1295, CERPOP, Inserm, Toulouse University, Toulouse, France; e Clinical Investigation Center 1436, Team PEPSS, Toulouse University Hospital, Inserm, Toulouse University, Toulouse, France

**Keywords:** Pregabalin, Gabapentin, Gabapentinoids, Prescription drug abuse, Prescription drug misuse, Pharmacoepidemiology

## Abstract

**Background:**

Following recent developments regarding gabapentinoid (i.e., pregabalin and gabapentin) abuse and misuse in France, this study aims to describe new users of gabapentinoids from 2017 to 2021, estimate the incidence of high-dose use, and identify the associated factors.

**Methods:**

We used the French National Health Data System. In this retrospective, population-based cohort study, we included new users of pregabalin and gabapentin, aged 18 years and older, in two separate cohorts. We defined new users as patients with at least three dispensings of either pregabalin or gabapentin within a 12-month period, with the first dispensing occurring from January 1, 2017 to December 31, 2021, and no dispensing in the previous 12 months. We used high-dose use as a proxy for potential abuse and misuse. We defined high-dose use as exposure to an average daily dose exceeding the maximum recommended dose. We followed patients for up to 24 months. Then, we calculated the cumulative incidence of high-dose use in each cohort. Finally, we identified factors associated with high-dose use in each cohort using Cox models.

**Findings:**

We included 898,887 new users of pregabalin and 271,832 new users of gabapentin. Since 2020, the monthly number of new users of pregabalin has decreased and the monthly number of new users of gabapentin has increased. In both cohorts, the median age was 64 years, and about 60% of patients were women. Incidence rates were 26.0 [95% CI: 25.8, 26.3] per 1000 person-years for high-dose use of pregabalin and 10.0 [9.7, 10.3] per 1000 person-years for high-dose use of gabapentin. During treatment episodes where high-dose use was identified, patients in the pregabalin cohort used higher daily doses than those in the gabapentin cohort. The main factors associated with high-dose use of pregabalin and gabapentin included prior exposure to the other gabapentinoid, prior exposure to opioid agonist treatments, prior exposure to strong opioid analgesics, younger age, being a man, paraplegia, active cancers, and higher number of prescribers.

**Interpretation:**

Prescribers should exercise extra caution when initiating gabapentinoids in patients at risk of high-dose use.

**Funding:**

This study is part of the METEOR study, which was funded by EPI-PHARE (https://www.epi-phare.fr/).

**Research in context**

**Evidence before this study**

We searched PubMed for articles published from database inception to July 11, 2025 using the terms (“pregabalin” OR “gabapentin” OR “gabapentinoid”) AND (“abuse” OR “misuse”) AND “France”. We did not apply any restrictions on language or study type. The search yielded 33 studies reporting increasing gabapentinoid abuse and misuse in France, especially from 2018 onward. However, the only population-based study assessing the patterns of gabapentinoid use in France was published in 2019, with data from 2006 to 2014, limiting understanding of the current situation.

**Added value of this study**

This study provides recent data to better understand the current patterns of gabapentinoid use in France. Using the French National Health Data System, we described new users of gabapentinoids from 2017 to 2021, estimated the incidence of high-dose use, and identified the associated factors.

**Implications of all the available evidence**

Prescribers should exercise extra caution when initiating gabapentinoids in patients at risk of high-dose use. Close monitoring is required to assess whether the increase in gabapentin use will also lead to an increase in abuse and misuse.

## Introduction

In Europe, gabapentinoids (i.e., pregabalin and gabapentin) are approved for epilepsy and neuropathic pain, with pregabalin also approved for generalized anxiety disorder. In most European countries, including France, pregabalin is more commonly used than gabapentin.^1^ Gabapentinoid use has markedly increased worldwide, with a more than four-fold increase from 2008 to 2018. In France, the average annual increase was over 5%, despite already having one of the highest baseline exposures across European countries. This increase is driven by both on- and off-label use, although limited evidence supports gabapentinoids’ efficacy outside the approved indications.^2^ This increase may be explained by the search for alternatives to opioids and benzodiazepines.^1^

Evidence of gabapentinoid abuse (i.e., intentional use to achieve desirable non-therapeutic effects, according to the ACTTION definition^3^) and misuse (i.e., intentional use for therapeutic purposes that deviates from the summary of product characteristics, according to the ACTTION definition^3^) has accumulated since 2010.^4^ Systematic reviews have highlighted that gabapentinoids are increasingly misused to self-medicate; that gabapentinoids can produce desirable non-therapeutic effects alone, but are commonly used concomitantly with other drugs; and that opioid use disorder is the main risk factor for gabapentinoid abuse.^4^^,^^5^ Gabapentinoid abuse typically involves supratherapeutic doses, often far exceeding the maximum recommended dose, to achieve euphoric effects.^4^, ^5^, ^6^ Additionally, gabapentinoid abuse and misuse are associated with patient harm, including hospital admission and opioid overdose death.^5^^,^^7^ In response, many countries have imposed restrictions on the prescribing and dispensing of pregabalin since 2015, and some have extended these restrictions to gabapentin.^6^

To date, the only population-based study assessing the patterns of gabapentinoid use in France found that high-dose use of pregabalin was twice as frequent as that of gabapentin from 2006 to 2014, and that high-dose use of pregabalin led to substance use disorders more often.^8^ These findings, along with data from the French Addictovigilance Network, prompted the French Medicines Agency to issue an alert to healthcare professionals in 2016 about the risk of gabapentinoid abuse and misuse.^9^ Since then, and especially from 2018 onward, reports of gabapentinoid abuse-related harms (i.e., hospital admissions due to severe neurological, psychiatric, or cardiac adverse reactions; requests for addiction care; and deaths) have increased, particularly for pregabalin.^6^^,^^10^ In this context, pregabalin was reclassified as a second-line treatment for neuropathic pain in the French guideline update of April 2020, while gabapentin was maintained as a first-line treatment.^11^ Eventually, the French Medicines Agency imposed restrictions on the prescribing and dispensing of pregabalin in May 2021,^12^ although those of gabapentin remained unchanged. The prescription duration is now limited to six months and requires a secure, tamper-resistant prescription form.^13^ To better understand the current patterns of gabapentinoid use in France given these recent developments, this study aims to describe new users of gabapentinoids from 2017 to 2021, estimate the incidence of high-dose use, and identify the associated factors. We used high-dose use as a proxy for potential abuse and misuse.

## Methods

### Data source

We used the French National Health Data System (SNDS). The SNDS covers more than 99% of the French population (i.e., 68 million inhabitants in 2024) regardless of socioeconomic and employment status. The SNDS is one of the largest healthcare databases in the world and is commonly used in pharmacoepidemiology.^14^

The SNDS compiles data prospectively recorded from the French Health Insurance Database for outpatient care, and from the French National Hospital Discharge Database for inpatient care. Healthcare consumers and professionals are assigned a unique, lifelong pseudonym. For reimbursed drugs dispensed in community pharmacies, the dispensing date and the number of boxes dispensed are recorded. Reimbursed drugs are classified according to the Anatomical Therapeutic Chemical classification system. Diagnoses recorded during hospital stays and long-term diseases are classified according to the International Statistical Classification of Diseases and Related Health Problems – Tenth Revision. Data regarding race, ethnicity, exposure to non-reimbursed drugs, and exposure to substances are unavailable in the SNDS. Further information on the SNDS content has been published previously.^15^

### Study population

In this retrospective, population-based cohort study, we included new users of pregabalin and gabapentin, aged 18 years and older, in two separate cohorts. We defined new users as patients with at least three dispensings of either pregabalin or gabapentin within a 12-month period, with the first dispensing occurring from January 1, 2017 to December 31, 2021, and no dispensing in the previous 12 months. The cohort entry date was the date of first dispensing of either pregabalin or gabapentin. Patients exposed to both pregabalin and gabapentin were included in both cohorts at their respective time points.

We excluded prevalent users to prevent bias because the risk of abuse and misuse is likely to vary over time.^8^ This is typically referred to as a new-user design.^16^ Additionally, we excluded patients with occasional use because abuse and misuse typically occur with chronic use. Finally, we excluded patients with unknown age and sex, and those with non-continuous health plan enrolment in the 12 months prior to cohort entry.

### Exposure

We estimated treatment episodes based on dispensing dates because the dose regimen or days’ supply are unavailable in the SNDS. We considered a gap of more than 35 days (i.e., four weeks plus a one-week grace period) between two consecutive dispensings as a treatment interruption because gabapentinoids can be dispensed for a maximum of four weeks in France. Following a treatment interruption, a new treatment episode begins with the subsequent dispensing and lasts until the final dispensing or the next treatment interruption. Finally, we calculated the average daily dose by dividing the total amount of either pregabalin or gabapentin dispensed within each treatment episode by the duration of the treatment episode. We expressed average daily doses in Defined Daily Doses (DDDs) for comparison between the two cohorts. The DDD of pregabalin is 300 mg and the DDD of gabapentin is 1800 mg. This method is commonly used in pharmacoepidemiology.^17^

### Outcome

We used high-dose use as a proxy for potential abuse and misuse. We defined high-dose use as exposure to an average daily dose exceeding the maximum recommended dose specified in the summary of product characteristics (i.e., 600 mg for pregabalin and 3600 mg for gabapentin, corresponding to 2 DDDs). Previous studies highlighted that gabapentinoid abuse typically involves supratherapeutic doses, often far exceeding the maximum recommended dose, to achieve euphoric effects.^4^, ^5^, ^6^

We followed patients for up to 24 months. We defined time-to-event as the time from cohort entry to the midpoint of the first treatment episode where high-dose use was identified. Time-to-event was censored at the time of death from any cause, loss to follow-up, or end of follow-up, whichever occurred first.

### Covariates

We extracted age and sex at cohort entry. We estimated socioeconomic status at cohort entry using the French Deprivation Index.^18^ This ecological index is derived from the first component of a principal component analysis of four socioeconomic variables based on the place of residence: median household income, proportion of high school graduates, proportion of blue-collar workers, and unemployment rate. Finally, the higher the index, the higher the deprivation.

We identified comorbidities at cohort entry using the disease mapping of the French Health Insurance.^19^ The mapping identifies diseases using algorithms based on diagnoses recorded during hospital stays, long-term diseases, and reimbursed drugs dispensed. We extracted data on the following comorbidities related to gabapentinoid indications or substance use disorders: psychiatric disorders (i.e., any psychiatric disorder, depression and mood disorders, substance use disorders, and psychotic disorders), neurological diseases (i.e., any neurological disease, dementia, Parkinson’s disease, epilepsy, paraplegia, multiple sclerosis, and myopathy or myasthenia), chronic inflammatory diseases (i.e., any chronic inflammatory disease, rheumatoid arthritis, and ankylosing spondylitis), and other comorbidities (i.e., neurocardiovascular diseases, diabetes, obesity, active cancers, disc disease, carpal tunnel syndrome, nerve root compression, chronic end-stage renal disease, spinal fracture, and migraine).

We defined prior exposure to the other gabapentinoid and prior exposure to other prescription drugs as having at least one dispensing in the 12 months prior to cohort entry. Regarding other prescription drugs, we identified the following prescription drugs according to the Anatomical Therapeutic Chemical classification system: weak opioid analgesics (i.e., codeine and paracetamol combinations, codeine and ibuprofen combinations, dihydrocodeine, tramadol, tramadol and paracetamol combinations, and opium and paracetamol combinations), strong opioid analgesics (i.e., morphine, oxycodone, oxycodone and naloxone combinations, fentanyl, hydromorphone, analgesic methadone, and analgesic buprenorphine), antiepileptics, antipsychotics, anxiolytics, hypnotics and sedatives, antidepressants, and opioid agonist treatments (i.e., methadone, buprenorphine, and buprenorphine and naloxone combinations).

### Statistical analysis

First, we described patient baseline sociodemographic characteristics (i.e., age, sex, and French Deprivation Index), patterns of exposure to gabapentinoids (i.e., initial prescriber, number of prescribers, number of treatment episodes, and duration of treatment episodes), baseline comorbidities, prior exposure to the other gabapentinoid, and prior exposure to other prescription drugs in each cohort. Additionally, we constructed time series of the monthly number of new users of gabapentinoids included in the two cohorts from January 2017 to December 2021 to estimate the trend in pregabalin and gabapentin initiation. We calculated the monthly number of new users per 100,000 inhabitants, using the population census from the French National Institute of Statistics and Economic Studies as the denominator. We applied a seasonal-trend decomposition procedure based on loess to identify trends in the time series.^20^

Then, we calculated the cumulative incidence (F(t)=1−S(t)) of high-dose use in each cohort. We estimated the survival function (S(t)) using the Kaplan–Meier estimator. We compared the survival distributions between the two cohorts using the log-rank test. Additionally, we calculated the incidence rates of high-dose use per 1000 person-years with 95% CIs.

Finally, we identified factors associated with high-dose use in each cohort using Cox models. We included patient baseline sociodemographic characteristics, patterns of exposure to gabapentinoids, baseline comorbidities, prior exposure to the other gabapentinoid, and prior exposure to other prescription drugs in the models (Supplementary Table S1). We selected these covariates a priori, informed by relevant literature and expert consultation. We excluded patients with missing data for any of these covariates from both models. We fitted continuous covariates as restricted cubic splines with five degrees of freedom to account for non-linear relationships between continuous covariates and the outcome. We tested the proportional hazard assumption using the Grambsch and Therneau test.^21^ Due to non-proportional hazards, we used a robust variance estimator and interpreted the model’s hazard ratios (HRs) as time-averaged HRs.^22^ Additionally, we estimated time-dependent HRs by plotting the smoothed, scaled Schoenfeld residuals over time.^21^

### Sensitivity analysis

We assessed the robustness of the findings using different exposure definitions. In addition to the 35-day threshold used in the main analysis, we calculated the average daily dose with 42-day (i.e., four weeks plus a two-week grace period) and 56-day (i.e., four weeks plus a four-week grace period) thresholds to define treatment interruptions. We assessed the effect on the cumulative incidence of high-dose use.

### Ethics

We conducted the study under the permanent access granted to hospital researchers, and as such, did not require specific authorization from the French Data Protection Authority. Given that the SNDS is a pseudonymized database, informed consent was not required. We registered the study protocol in the European Medicines Agency’s Catalogue of Real-World Data Studies.^23^ The reporting of the study adheres to the Strengthening the Reporting of Observational Studies in Epidemiology statement.^24^

We conducted analyses using Oracle Database 19c Enterprise Edition, R version 4.3.3,^25^ and the survival package version 3.5-8.^26^ The code is available at https://gitlab.com/soeiro/meteor.

### Role of the funding source

The funder of the study had no role in study design, data collection, data analysis, data interpretation, writing of the manuscript, or decision to submit the manuscript for publication.

## Results

### Description of new users of gabapentinoids

**Fig. 1:**
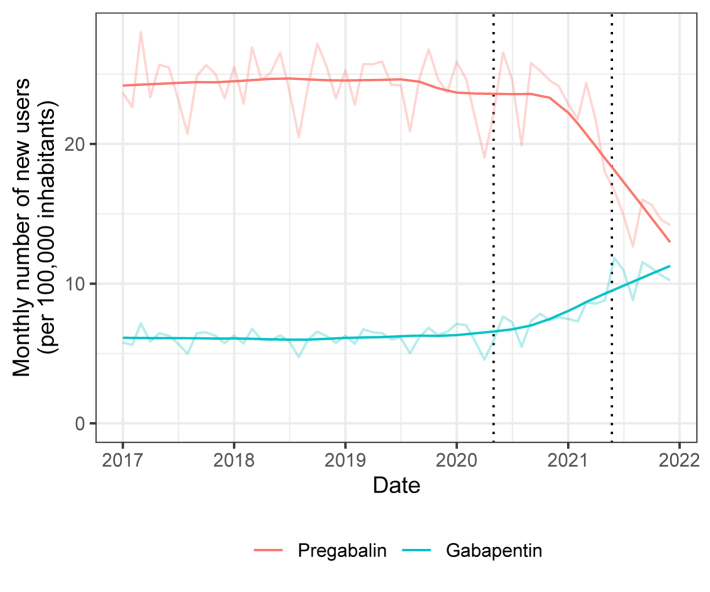
Trend in pregabalin and gabapentin initiation in France from 2017 to 2021, based on the monthly number of new users of gabapentinoids included in the two cohorts. The first dotted line corresponds to April 2020, when pregabalin was reclassified as a second-line treatment for neuropathic pain in the French guideline update. The second dotted line corresponds to May 2021, when the French Medicines Agency imposed restrictions on the prescribing and dispensing of pregabalin.

From January 1, 2017 to December 31, 2021, 4,113,310 patients were dispensed pregabalin and 1,522,177 patients were dispensed gabapentin. Among them, we included 898,887 new users of pregabalin and 271,832 new users of gabapentin. Since 2020, the monthly number of new users of pregabalin has decreased (from 23.7 new users per 100,000 inhabitants in December 2019 to 14.2 new users per 100,000 inhabitants in December 2021) and the monthly number of new users of gabapentin has increased (from 6.6 new users per 100,000 inhabitants in December 2019 to 10.2 new users per 100,000 inhabitants in December 2021) (Fig. 1).

In both cohorts, the median age was 64 years, and about 60% of patients were women (Table 1). Pregabalin was most commonly initiated by general practitioners (63.1%, n = 549,865), followed by hospital practitioners (14.8%, n = 129,440), rheumatologists (4.7%, n = 40,755), neurologists (3.9%, n = 33,733), and psychiatrists (2.0%, n = 17,642). To a lesser extent, gabapentin was most commonly initiated by general practitioners (51.3%, n = 135,168), followed by hospital practitioners (18.6%, n = 48,867), neurologists (11.2%, n = 29,606), rheumatologists (4.4%, n = 11,480), and psychiatrists (1.1%, n = 2924). In both cohorts, diabetes (pregabalin: 22.1%, n = 194,644; gabapentin: 21.5%, n = 57,326) and active cancers (pregabalin: 11.8%, n = 103,892; gabapentin: 9.8%, n = 26,025) were common, while epilepsy (pregabalin: 2.1%, n = 18,391; gabapentin: 2.6%, n = 7008) was not. About 4% of patients had substance use disorders.

**Table 1:** Baseline characteristics and patterns of exposure to gabapentinoids in new users of pregabalin and gabapentin in France from 2017 to 2021.

	Pregabalin	Gabapentin
Number of new users, n	898,887	271,832
Age, year, median [IQR]	64.0 [51.0, 75.0]	64.0 [51.0, 75.0]
Sex, n (%)		
Women	519,074 (57.7)	160,122 (58.9)
Men	379,813 (42.3)	111,710 (41.1)
French deprivation index, median, [IQR]	0.4 [-0.7, 1.3]	0.4 [-0.7, 1.4]
Missing	51,449	13,308
French deprivation index, n (%)		
1st quintile (least deprived)	129,958 (15.3)	42,463 (16.4)
2nd quintile	151,414 (17.9)	45,975 (17.8)
3rd quintile	173,437 (20.5)	50,333 (19.5)
4th quintile	188,676 (22.3)	54,661 (21.1)
5th quintile (most deprived)	203,953 (24.1)	65,092 (25.2)
Missing	51,449	13,308
Initial prescriber, n (%)		
General practitioner	549,865 (63.1)	135,168 (51.3)
Hospital practitioner	129,440 (14.8)	48,867 (18.6)
Neurologist	33,733 (3.9)	29,606 (11.2)
Rheumatologist	40,755 (4.7)	11,480 (4.4)
Psychiatrist	17,642 (2.0)	2924 (1.1)
Orthopaedic surgeon	15,382 (1.8)	4210 (1.6)
Other prescriber	85,075 (9.8)	31,114 (11.8)
Missing	26,995	8463
Number of prescribers, median [IQR]	2.0 [1.0, 2.0]	2.0 [1.0, 2.0]
Number of treatment episodes, median [IQR]	4.0 [2.0, 6.0]	4.0 [2.0, 7.0]
Duration of treatment episodes, days, median [IQR]	49.5 [35.0, 76.0]	49.0 [35.0, 77.0]
Psychiatric disorders, n (%)		
Any psychiatric disorder	132,697 (15.0)	41,006 (15.4)
Depression and mood disorders	57,761 (6.6)	18,158 (6.8)
Substance use disorders	39,462 (4.5)	11,324 (4.2)
Psychotic disorders	12,856 (1.5)	3496 (1.3)
Missing	17,096	5364
Neurological diseases, n (%)		
Any neurological disease	90,852 (10.3)	38,878 (14.6)
Dementia	25,533 (2.9)	10,132 (3.8)
Parkinson’s disease	17,909 (2.0)	8134 (3.1)
Epilepsy	18,391 (2.1)	7008 (2.6)
Paraplegia	12,041 (1.4)	5093 (1.9)
Multiple sclerosis	9296 (1.1)	5093 (1.9)
Myopathy or myasthenia	2092 (0.2)	953 (0.4)
Missing	17,096	5364
Chronic inflammatory diseases, n (%)		
Any chronic inflammatory disease	43,766 (5.0)	14,761 (5.5)
Rheumatoid arthritis	15,278 (1.7)	4915 (1.8)
Ankylosing spondylitis	11,881 (1.3)	4173 (1.6)
Missing	17,096	
Other comorbidities, n (%)		
Neurocardiovascular diseases	239,005 (27.1)	73,038 (27.4)
Diabetes	194,644 (22.1)	57,326 (21.5)
Obesity	117,927 (13.4)	39,570 (14.8)
Active cancers	103,892 (11.8)	26,025 (9.8)
Disc disease	20,311 (2.3)	5906 (2.2)
Carpal tunnel syndrome	10,703 (1.2)	3157 (1.2)
Nerve root compression	9919 (1.1)	2537 (1.0)
Chronic end-stage renal disease	9703 (1.1)	2800 (1.1)
Spinal fracture	4999 (0.6)	1276 (0.5)
Migraine	2616 (0.3)	952 (0.4)
Missing	17,096	5364
Prior exposure to the other gabapentinoid, n (%)		
Pregabalin	NA	78,825 (29.0)
Gabapentin	39,876 (4.4)	NA
Prior exposure to other prescription drugs, n (%)		
Weak opioid analgesics	543,312 (60.4)	165,588 (60.9)
Strong opioid analgesics	114,350 (12.7)	39,971 (14.7)
Antiepileptics	49,330 (5.5)	21,978 (8.1)
Antipsychotics	56,946 (6.3)	16,435 (6.0)
Anxiolytics	371,788 (41.4)	114,647 (42.2)
Hypnotics and sedatives	180,577 (20.1)	56,138 (20.7)
Antidepressants	284,415 (31.6)	103,857 (38.2)
Opioid agonist treatments	6213 (0.7)	1199 (0.4)

NA: not applicable.

In the pregabalin cohort, 4.4% (n = 39,876) of patients had prior exposure to gabapentin. Conversely, in the gabapentin cohort, 29.0% (n = 78,825) of patients had prior exposure to pregabalin. Regarding other prescription drugs, prior exposure to weak opioid analgesics (pregabalin: 60.4%, n = 543,312; gabapentin: 60.9%, n = 165,588) and anxiolytics (pregabalin: 41.4%, n = 371,788; gabapentin: 42.2%, n = 114,647) were the most common.

### Incidence of high-dose use of gabapentinoids

**Fig. 2:**
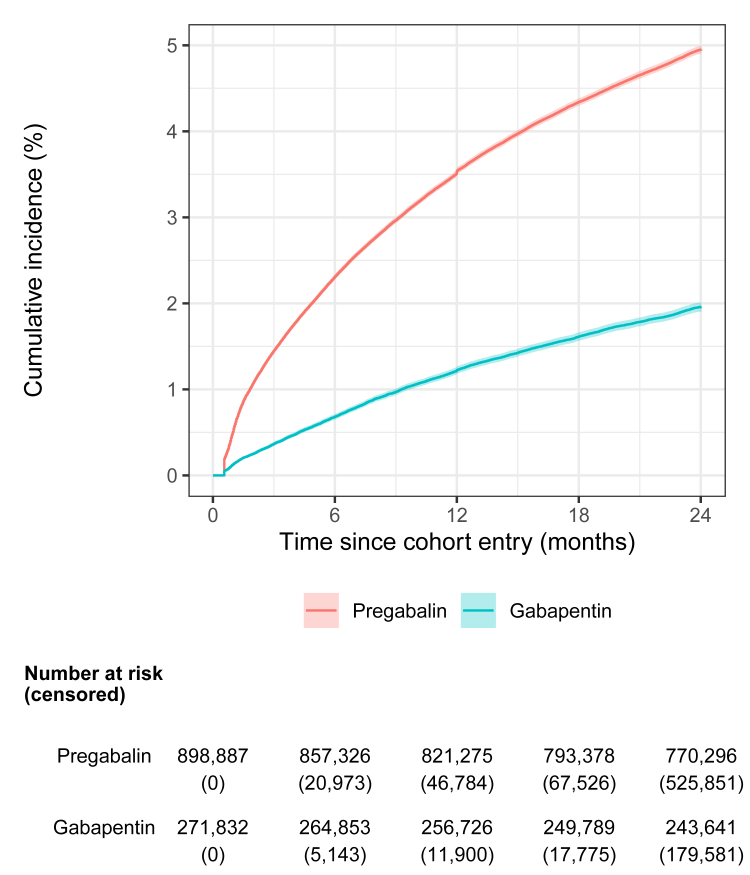
Cumulative incidence of high-dose use of pregabalin and gabapentin (log-rank test: p < 0.0001).

During the 2,167,523 person-years of follow-up, 4.8% (n = 42,992) of new users of pregabalin and 1.9% (n = 5134) of new users of gabapentin used high doses (log-rank test: p < 0.0001) (Fig. 2). This resulted in incidence rates of 26.0 [95% CI: 25.8, 26.3] per 1000 person-years for high-dose use of pregabalin and 10.0 [9.7, 10.3] per 1000 person-years for high-dose use of gabapentin. The sensitivity analysis revealed similar patterns with 42-day and 56-day thresholds to define treatment interruptions, with lower incidence rates (Supplementary Fig. S1).

### Factors associated with high-dose use of gabapentinoids

During treatment episodes where high-dose use was identified, patients in the pregabalin cohort used higher daily doses than those in the gabapentin cohort (median: 2.4 [IQR: 2.2, 3.2] DDDs for pregabalin versus 2.3 [2.1, 2.5] DDDs for gabapentin). In both cohorts, patients with high-dose use also had more treatment episodes (5.0 [3.0, 8.0] versus 3.0 [2.0, 6.0] for pregabalin, and 6.0 [4.0, 8.0] versus 4.0 [2.0, 7.0] for gabapentin), longer treatment episodes (55.5 [35.0, 82.5] days versus 49.0 [35.0, 75.0] days for pregabalin, and 58.0 [35.0, 85.0] days versus 49.0 [35.0, 77.0] days for gabapentin), and more prescribers (2.0 [2.0, 3.0] versus 2.0 [1.0, 2.0] in both cohorts), up to several dozen for pregabalin (Supplementary Table S2).

Additionally, patients with high-dose use were younger (52.0 [40.0, 63.0] years versus 64.0 [52.0, 76.0] years for pregabalin, and 54.0 [44.0, 66.0] years versus 64.0 [52.0, 76.0] years for gabapentin). The proportion of men was higher in patients with high-dose use (pregabalin: 56.1%, n = 24,122 versus 41.6%, n = 355,691; gabapentin: 49.3%, n = 2529 versus 40.9%, n = 109,181). Prior exposure to the other gabapentinoid (pregabalin: 8.1%, n = 3483 versus 4.3%, n = 36,393; gabapentin: 41.3%, n = 2120 versus 28.8%, n = 76,705), prior exposure to strong opioid analgesics (pregabalin: 15.9%, n = 6815 versus 12.6%, n = 107,535; gabapentin: 23.0%, n = 1181 versus 14.5%, n = 38,790), and prior exposure to opioid agonist treatments (pregabalin: 5.2%, n = 2244 versus 0.5%, n = 3969; gabapentin: 1.2%, n = 60 versus 0.4%, n = 1139) were more common in patients with high-dose use.

**Fig. 3:**
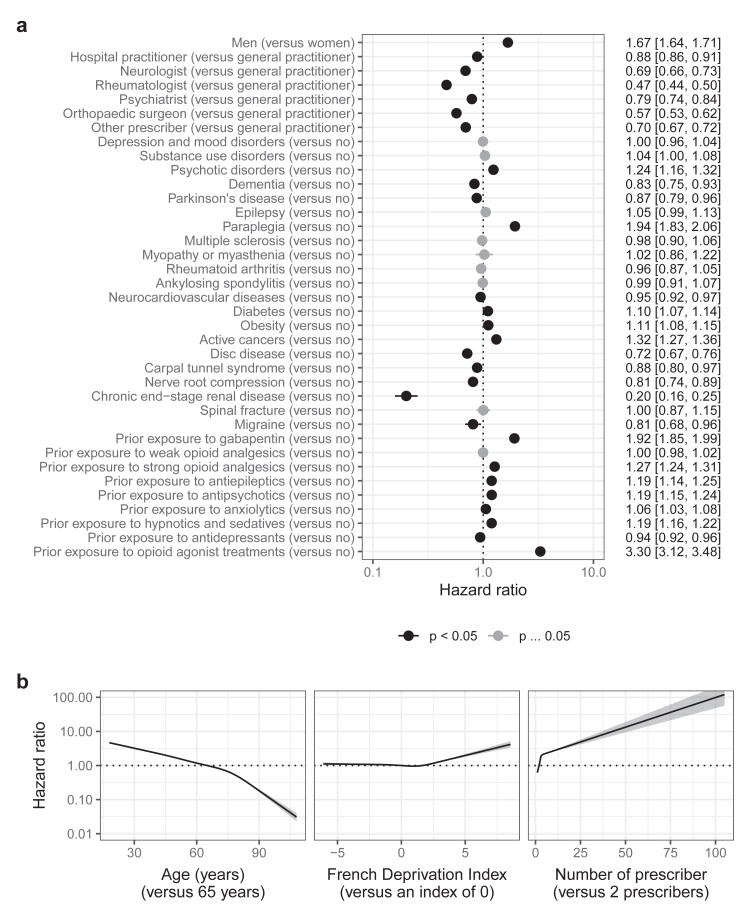
Factors associated with high-dose use of pregabalin. a) categorical covariates. b) continuous covariates. The dotted lines correspond to a null effect. For the French Deprivation Index, the higher the index, the higher the deprivation.

In the pregabalin cohort, the main factors associated with high-dose use were prior exposure to opioid agonist treatments (HR: 3.30 [95% CI: 3.12, 3.48]), paraplegia (1.94 [1.83, 2.06]), prior exposure to gabapentin (1.92 [1.85, 1.99]), being a man (1.67 [1.64, 1.71]), active cancers (1.32 [1.27, 1.36]), and prior exposure to strong opioid analgesics (1.27 [1.24, 1.31]) (Fig. 3 and Supplementary Table S3). To a lesser extent, psychotic disorders and prior exposure to other prescription drugs were also associated with high-dose use. Conversely, initial prescription by specialists was associated with lower risk of high-dose use. Higher number of prescribers, younger age, and higher deprivation were non-linearly associated with high-dose use. The risk of high-dose use increased during follow-up for active cancers (Supplementary Fig. S2). Conversely, the risk of high-dose use decreased during follow-up for prior exposure to opioid agonist treatments and prior exposure to gabapentin.

**Fig. 4:**
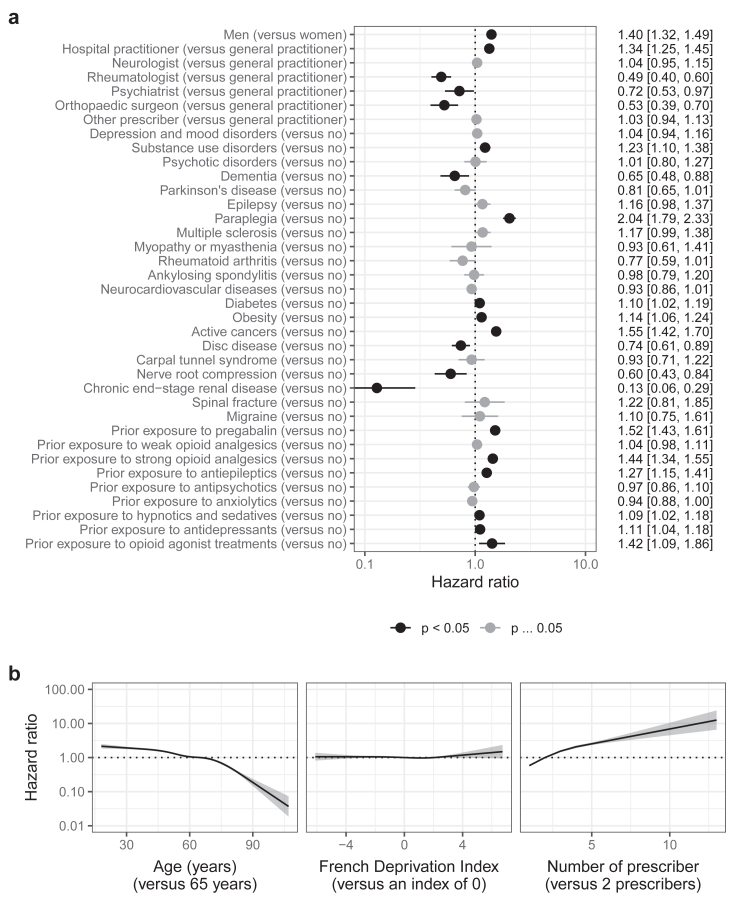
Factors associated with high-dose use of gabapentin. a) categorical covariates. b) continuous covariates. The dotted lines correspond to a null effect. For the French Deprivation Index, the higher the index, the higher the deprivation.

Similarly, in the gabapentin cohort, the main factors associated with high-dose use were paraplegia (2.04 [1.79, 2.33]), active cancers (1.55 [1.42, 1.70]), prior exposure to pregabalin (1.52 [1.43, 1.61]), prior exposure to strong opioid analgesics (1.44 [1.34, 1.55]), prior exposure to opioid agonist treatments (1.42 [1.09, 1.86]), and being a man (1.40 [1.32, 1.49]) (Fig. 4 and Supplementary Table S3). To a lesser extent, initial prescription by hospital practitioners, prior exposure to antiepileptics, and substance use disorders were also associated with high-dose use. Conversely, initial prescription by rheumatologists, orthopaedic surgeons, and psychiatrists was associated with lower risk of high-dose use. Higher number of prescribers and younger age were non-linearly associated with high-dose use. The risk of high-dose use increased during follow-up for prior exposure to pregabalin (Supplementary Fig. S3). Conversely, the risk of high-dose use decreased during follow-up for paraplegia and prior exposure to strong opioid analgesics.

## Discussion

To better understand the current patterns of gabapentinoid use in France, this study aims to describe new users of gabapentinoids from 2017 to 2021, estimate the incidence of high-dose use, and identify the associated factors.

First, the findings suggest that gabapentinoids are commonly used for neuropathic pain (e.g., diabetic neuropathy) in France, but not for epilepsy and generalized anxiety disorder. We found that more than 20% of patients had diabetes, and less than 3% had epilepsy. Additionally, gabapentinoids were most commonly initiated by general practitioners, particularly pregabalin. Gabapentin was initiated by neurologists in 11% of patients, while this was less common for pregabalin. Finally, pregabalin was initiated by psychiatrists for only 2% of patients. Similarly, gabapentinoids are most commonly used for pain in the USA, with widespread off-label use.^2^ In Germany, 74% of patients on gabapentinoids were either not diagnosed with neuropathic pain or were diagnosed with pain without evidence of gabapentinoid efficacy.^27^

Second, we found that high-dose use of pregabalin was more than twice as frequent as that of gabapentin and involved higher daily doses. The main factors associated with high-dose use of pregabalin and gabapentin included prior exposure to the other gabapentinoid, prior exposure to opioid agonist treatments, prior exposure to strong opioid analgesics, younger age, being a man, paraplegia, active cancers, and higher number of prescribers. Regarding the use of gabapentinoids and opioids, extra caution is required when initiating gabapentinoids in patients already exposed to opioids due to the risk of opioid overdose death.^28^

These findings suggest several hypotheses about the reasons for high-dose use of gabapentinoids. First, some patients may use higher doses due to insufficient efficacy, particularly in the case of off-label use or a switch from the other gabapentinoid. Second, some patients may develop pharmacological tolerance, requiring higher doses to achieve the initial effect. Third, some patients may use higher doses to achieve desirable non-therapeutic effects. Previous studies highlighted that gabapentinoid abuse typically involves supratherapeutic doses, often far exceeding the maximum recommended dose, to achieve euphoric effects.^4^, ^5^, ^6^ Finally, some patients may doctor shop for gabapentinoids, including for trafficking purposes, given the extremely high number of prescribers for some patients. Pregabalin was the fifth most commonly doctor-shopped prescription drug in France in 2019, with a marked increase compared to previous years.^29^

To date, the only population-based study assessing the patterns of gabapentinoid use in France found that 10.5% of new users of pregabalin and 5.8% of new users of gabapentin used high doses during the first two years of follow-up, from 2006 to 2014.^8^ We found that 4.8% of new users of pregabalin and 1.9% of new users of gabapentin used high doses from 2017 to 2021. External comparison is challenging due to methodological differences between the studies (e.g., data sources and exposure definitions), in addition to differences in the study period. However, both studies found that high-dose use of pregabalin was about twice as frequent as that of gabapentin and identified similar associated factors. Similarly, 8.5% of new users of pregabalin in Sweden and 6.5% in Denmark used high doses from 2006 to 2009, but data were unavailable for gabapentin.^30^^,^^31^ Once again, the factors associated with high-dose use of pregabalin were similar. In the USA, high-dose use of pregabalin is also more frequent than that of gabapentin, although gabapentin is more commonly used than pregabalin.^32^

Finally, although pregabalin remains more commonly used than gabapentin in France, a shift toward increased gabapentin initiation has occurred since the reclassification of pregabalin as a second-line treatment for neuropathic pain in May 2020 and the restrictions on the prescribing and dispensing of pregabalin in May 2021. This suggests that prescribers may now prefer initiating or switching to gabapentin. An interrupted time-series analysis found that the restrictions on the prescribing and dispensing of pregabalin resulted in a decrease in pregabalin use in France from June 2020 to May 2022, without a decrease in high-dose use.^13^ Similarly, many countries have imposed restrictions on the prescribing and dispensing of pregabalin since 2015, including in the Middle East (e.g., Saudi Arabia, United Arab Emirates, Turkey, and Jordan), the USA, and the UK.^6^ In the UK, gabapentinoid use has been extensively studied since their reclassification as scheduled drugs in April 2019. A study using the OpenPrescribing data found that gabapentinoid use increased from 1.5% to 11.9% annually from 2015 to 2020, with the increase slowing since 2019.^33^ A study using the Clinical Practice Research Datalink found that gabapentin initiation has decreased since 2016–2017 and pregabalin initiation since 2018–2019.^34^ However, several studies found that the reclassification had little impact on continued gabapentinoid use in prevalent users and on the prescribing habits of general practitioners.^34^^,^^35^

### Limitations

As with any study assessing prescription drug abuse and misuse using health insurance data, the main limitation is the lack of a gold standard measure.^36^ We used high-dose use as a proxy for potential abuse and misuse, but this does not capture all patterns of abuse and misuse (e.g., doctor shopping). Additionally, we estimated treatment episodes based on dispensing dates because the dose regimen or days’ supply are unavailable in the SNDS. Therefore, residual misclassification of patients cannot be excluded. However, the findings remained robust in the sensitivity analysis. Finally, although we adjusted the models for multiple covariates, another limitation is that residual confounding (e.g., pain severity and concomitant exposure to other prescription drugs) may have biased the findings.

### Conclusion

High-dose use of pregabalin is more than twice as frequent as that of gabapentin and involves higher daily doses. The main factors associated with high-dose use of pregabalin and gabapentin include prior exposure to the other gabapentinoid, prior exposure to opioid agonist treatments, prior exposure to strong opioid analgesics, younger age, being a man, paraplegia, active cancers, and higher number of prescribers. Prescribers should exercise extra caution when initiating gabapentinoids in patients at risk of high-dose use. Finally, given the shift toward increased gabapentin initiation, close monitoring is required to assess whether the increase in gabapentin use will also lead to an increase in abuse and misuse.

## Contributors

• Thomas Soeiro: conceptualization, data curation, formal analysis, funding acquisition, methodology, project administration, software, visualization, writing – original draft, writing – review & editing
• Marina Uras: data curation, formal analysis, software
• Émilie Jouanjus: writing – original draft, writing – review & editing
• Maryse Lapeyre-Mestre: conceptualization, methodology, writing – review & editing
• Joëlle Micallef: conceptualization, funding acquisition, supervision, writing – review & editing

Thomas Soeiro and Marina Uras accessed and verified the data. The other authors did not have the required authorisation to access the data. All authors had final responsibility for the decision to submit the manuscript for publication.

## Data sharing statement

According to French law, we cannot share data from the French National Health Data System. However, any individual or organization can access the data to conduct studies of public interest, upon authorization from the French Data Protection Authority.

## Declaration of interests

We declare no competing interests.
