# Supplementary material for: High-dose use of pregabalin and gabapentin in France: a retrospective, population-based cohort study
Source: Lancet Reg Health Eur. 2025 Aug 7;57:101424. doi: 10.1016/j.lanepe.2025.101424 (PMC12355116; doi:10.1016/j.lanepe.2025.101424)

Patterns of pregabalin and gabapentin use in France: a retrospective, population-based cohort study

Thomas Soeiro, PharmD [1,2], Marina Uras, MSc [2], Émilie Jouanjus, PharmD [1,3,4], Maryse Lapeyre-Mestre, MD [3,5], Joëlle Micallef, MD [1,2]

1. UMR 1106, INS, Inserm, Aix-Marseille University, Marseille, France
2. Department of Clinical Pharmacology and Drug Surveillance, Marseille University Hospital, Marseille, France
3. Department of Medical and Clinical Pharmacology, Toulouse University Hospital, Toulouse, France
4. UMR 1295, CERPOP, Inserm, Toulouse University, Toulouse, France
5. Clinical Investigation Center 1436, Team PEPSS, Toulouse University Hospital, Inserm, Toulouse University, Toulouse, France

Corresponding author

Thomas Soeiro
thomas.soeiro@ap-hm.fr
Service de Pharmacologie Clinique et Pharmacosurveillance
Assistance Publique – Hôpitaux de Marseille
Hôpital Sainte Marguerite, Pavillon 4, 1er étage
270 boulevard de Sainte Marguerite, 13009 Marseille
+33 4 91 74 50 15

ORCID

- Thomas Soeiro: 0000-0003-2604-9673
- Émilie Jouanjus: 0000-0002-3510-2475
- Maryse Lapeyre-Mestre: 0000-0002-5494-5873
- Joëlle Micallef: 0000-0002-7172-7835

Keywords: pregabalin; gabapentin; gabapentinoids; prescription drug misuse; pharmacoepidemiology

Supplementary Table 1. Covariates included in the Cox models

**Covariate**	**Reference**
Age	65 years
Sex	Women
French Deprivation Index	Index of 0
Initial prescriber	General practitioner
Number of prescribers	2 prescribers
Depression and mood disorders	No
Substance use disorders	No
Psychotic disorders	No
Dementia	No
Parkinson’s disease	No
Epilepsy	No
Paraplegia	No
Multiple sclerosis	No
Myopathy or myasthenia	No
Rheumatoid arthritis	No
Ankylosing spondylitis	No
Neurocardiovascular diseases	No
Diabetes	No
Obesity	No
Active cancers	No
Disc disease	No
Carpal tunnel syndrome	No
Nerve root compression	No
Chronic end-stage renal disease	No
Spinal fracture	No
Migraine	No
Prior exposure to pregabalin	No
Prior exposure to gabapentin	No
Prior exposure to weak opioid analgesics	No
Prior exposure to strong opioid analgesics	No
Prior exposure to antiepileptics	No
Prior exposure to antipsychotics	No
Prior exposure to anxiolytics	No
Prior exposure to hypnotics and sedatives	No
Prior exposure to antidepressants	No
Prior exposure to opioid agonist treatments	No

Supplementary Table 2. Baseline characteristics and patterns of exposure to gabapentinoids in new users of pregabalin and gabapentin in France from 2017 to 2021, according high-dose use. NA: not applicable

	Pregabalin		Gabapentin	
	**High-dose use**	**No high-dose use**	**High-dose use**	**No high-dose use**
**Number of new users, n**	42992	855895	5134	266698
**Age, year, median [IQR]**	52.0 [40.0, 63.0]	64.0 [52.0, 76.0]	54.0 [44.0, 66.0]	64.0 [52.0, 76.0]
**Sex, n (%)**				
**Women**	18870 (43.9)	500204 (58.4)	2605 (50.7)	157517 (59.1)
**Men**	24122 (56.1)	355691 (41.6)	2529 (49.3)	109181 (40.9)
**French Deprivation Index, median, [IQR]**	0.4 [-0.7, 1.4]	0.4 [-0.7, 1.3]	0.3 [-0.7, 1.4]	0.4 [-0.7, 1.4]
**Missing**	2212	49237	266	13042
**French Deprivation Index, n (%)**				
**1st quintile (least deprived)**	6274 (15.4)	123684 (15.3)	828 (17.0)	41635 (16.4)
**2nd quintile**	7340 (18.0)	144074 (17.9)	889 (18.3)	45086 (17.8)
**3rd quintile**	8279 (20.3)	165158 (20.5)	949 (19.5)	49384 (19.5)
**4th quintile**	8460 (20.7)	180216 (22.3)	988 (20.3)	53673 (21.2)
**5th quintile (most deprived)**	10427 (25.6)	193526 (24.0)	1214 (24.9)	63878 (25.2)
**Missing**	2212	49237	266	13042
**Initial prescriber, n (%)**				
**General practitioner**	26374 (63.1)	523491 (63.1)	2019 (41.0)	133149 (51.5)
**Hospital practitioner**	7096 (17.0)	122344 (14.7)	1397 (28.3)	47470 (18.4)
**Neurologist**	1547 (3.7)	32186 (3.9)	608 (12.3)	28998 (11.2)
**Rheumatologist**	1059 (2.5)	39696 (4.8)	100 (2.0)	11380 (4.4)
**Psychiatrist**	1299 (3.1)	16343 (2.0)	48 (1.0)	2876 (1.1)
**Orthopaedic surgeon**	647 (1.5)	14735 (1.8)	52 (1.1)	4158 (1.6)
**Other prescriber**	3764 (9.0)	81311 (9.8)	704 (14.3)	30410 (11.8)
**Missing**	1206	25789	206	8257
**Number of prescribers, median [IQR]**	2.0 [2.0, 3.0]	2.0 [1.0, 2.0]	2.0 [2.0, 3.0]	2.0 [1.0, 2.0]
**Number of treatment episodes, median [IQR]**	5.0 [3.0, 8.0]	3.0 [2.0, 6.0]	6.0 [4.0, 8.0]	4.0 [2.0, 7.0]
**Duration of treatment episodes, days, median [IQR]**	55.5 [35.0, 82.5]	49.0 [35.0, 75.0]	58.0 [35.0, 85.0]	49.0 [35.0, 77.0]
**Psychiatric disorders, n (%)**				
**Any psychiatric disorder**	9185 (21.9)	123512 (14.7)	1079 (21.5)	39927 (15.3)

Depression and mood disorders	3527 (8.4)	54234 (6.5)	468 (9.3)	17690 (6.8)
Substance use disorders	3731 (8.9)	35731 (4.3)	396 (7.9)	10928 (4.2)
Psychotic disorders	1507 (3.6)	11349 (1.4)	93 (1.9)	3403 (1.3)
Missing	1029	16067	114	5250
**Neurological diseases, n (%)**				
Any neurological disease	4928 (11.7)	85924 (10.2)	966 (19.2)	37912 (14.5)
Dementia	370 (0.9)	25163 (3.0)	49 (1.0)	10083 (3.9)
Parkinson's disease	453 (1.1)	17456 (2.1)	91 (1.8)	8043 (3.1)
Epilepsy	1280 (3.1)	17111 (2.0)	195 (3.9)	6813 (2.6)
Paraplegia	1527 (3.6)	10514 (1.3)	302 (6.0)	4791 (1.8)
Multiple sclerosis	644 (1.5)	8652 (1.0)	173 (3.4)	4920 (1.9)
Myopathy or myasthenia	139 (0.3)	1953 (0.2)	25 (0.5)	928 (0.4)
Missing	1029	16067	114	5250
**Chronic inflammatory diseases, n (%)**				
Any chronic inflammatory disease	1871 (4.5)	41895 (5.0)	281 (5.6)	14480 (5.5)
Rheumatoid arthritis	478 (1.1)	14800 (1.8)	58 (1.2)	4857 (1.9)
Ankylosing spondylitis	670 (1.6)	11211 (1.3)	101 (2.0)	4072 (1.6)
Missing	1029	16067	114	5250
**Other comorbidities, n (%)**				
Neurocardiovascular diseases	7975 (19.0)	231030 (27.5)	1070 (21.3)	71968 (27.5)
Diabetes	7881 (18.8)	186763 (22.2)	971 (19.3)	56355 (21.6)
Obesity	5950 (14.2)	111977 (13.3)	895 (17.8)	38675 (14.8)
Active cancers	4823 (11.5)	99069 (11.8)	632 (12.6)	25393 (9.7)
Disc disease	1046 (2.5)	19265 (2.3)	122 (2.4)	5784 (2.2)
Carpal tunnel syndrome	444 (1.1)	10259 (1.2)	55 (1.1)	3102 (1.2)
Nerve root compression	485 (1.2)	9434 (1.1)	38 (0.8)	2499 (1.0)
Chronic end-stage renal disease	72 (0.2)	9631 (1.1)	7 (0.1)	2793 (1.1)
Spinal fracture	234 (0.6)	4765 (0.6)	27 (0.5)	1249 (0.5)
Migraine	158 (0.4)	2458 (0.3)	29 (0.6)	923 (0.4)
Missing	1029	16067	114	5250
**Prior exposure to the other gabapentinoid, n (%)**				
Pregabalin	NA	NA	2120 (41.3)	76705 (28.8)

**Gabapentin**	3483 (8.1)	36393 (4.3)	NA	NA
**Prior exposure to other prescription drugs, n (%)**				
**Weak opioid analgesics**	26882 (62.5)	516430 (60.3)	3381 (65.9)	162207 (60.8)
**Strong opioid analgesics**	6815 (15.9)	107535 (12.6)	1181 (23.0)	38790 (14.5)
**Antiepileptics**	3641 (8.5)	45689 (5.3)	628 (12.2)	21350 (8.0)
**Antipsychotics**	4825 (11.2)	52121 (6.1)	380 (7.4)	16055 (6.0)
**Anxiolytics**	19972 (46.5)	351816 (41.1)	2308 (45.0)	112339 (42.1)
**Hypnotics and sedatives**	10306 (24.0)	170271 (19.9)	1240 (24.2)	54898 (20.6)
**Antidepressants**	14565 (33.9)	269850 (31.5)	2349 (45.8)	101508 (38.1)
**Opioid agonist treatments**	2244 (5.2)	3969 (0.5)	60 (1.2)	1139 (0.4)

Supplementary Table 3. Categorical covariates associated with high-dose use of pregabalin and gabapentin. NA: not applicable

	Pregabalin		Gabapentin	
	**Crude HR [95% CI]**	**Adjusted HR [95% CI]**	**Crude HR [95% CI]**	**Adjusted HR [95% CI]**
**Men (versus women)**	0.59 [0.57, 0.61]	1.67 [1.64, 1.71]	0.35 [0.30, 0.41]	1.40 [1.32, 1.49]
**Hospital practitioner (versus general practitioner)**	0.18 [0.15, 0.20]	0.88 [0.86, 0.91]	0.68 [0.61, 0.75]	1.34 [1.25, 1.45]
**Neurologist (versus general practitioner)**	-0.06 [-0.11, -0.01]	0.69 [0.66, 0.73]	0.30 [0.21, 0.39]	1.04 [0.95, 1.15]
**Rheumatologist (versus general practitioner)**	-0.65 [-0.71, -0.58]	0.47 [0.44, 0.50]	-0.57 [-0.77, -0.36]	0.49 [0.40, 0.60]
**Psychiatrist (versus general practitioner)**	0.43 [0.37, 0.48]	0.79 [0.74, 0.84]	0.10 [-0.19, 0.39]	0.72 [0.53, 0.97]
**Orthopaedic surgeon (versus general practitioner)**	-0.16 [-0.23, -0.08]	0.57 [0.53, 0.62]	-0.21 [-0.49, 0.06]	0.53 [0.39, 0.70]
**Other prescriber (versus general practitioner)**	-0.04 [-0.07, -0.00]	0.70 [0.67, 0.72]	0.44 [0.35, 0.53]	1.03 [0.94, 1.13]
**Depression and mood disorders (versus no)**	0.28 [0.24, 0.31]	1.00 [0.96, 1.04]	0.35 [0.25, 0.44]	1.04 [0.94, 1.16]
**Substance use disorders (versus no)**	0.77 [0.74, 0.81]	1.04 [1.00, 1.08]	0.68 [0.58, 0.78]	1.23 [1.10, 1.38]
**Psychotic disorders (versus no)**	0.97 [0.92, 1.02]	1.24 [1.16, 1.32]	0.37 [0.16, 0.57]	1.01 [0.80, 1.27]
**Dementia (versus no)**	-1.14 [-1.24, -1.04]	0.83 [0.75, 0.93]	-1.27 [-1.55, -0.99]	0.65 [0.48, 0.88]
**Parkinson's disease (versus no)**	-0.64 [-0.73, -0.55]	0.87 [0.79, 0.96]	-0.52 [-0.73, -0.31]	0.81 [0.65, 1.01]
**Epilepsy (versus no)**	0.45 [0.39, 0.50]	1.05 [0.99, 1.13]	0.44 [0.30, 0.59]	1.16 [0.98, 1.37]
**Paraplegia (versus no)**	1.05 [1.00, 1.10]	1.94 [1.83, 2.06]	1.21 [1.10, 1.33]	2.04 [1.79, 2.33]
**Multiple sclerosis (versus no)**	0.36 [0.28, 0.44]	0.98 [0.90, 1.06]	0.59 [0.44, 0.74]	1.17 [0.99, 1.38]
**Myopathy or myasthenia (versus no)**	0.35 [0.18, 0.51]	1.02 [0.86, 1.22]	0.33 [-0.06, 0.73]	0.93 [0.61, 1.41]
**Rheumatoid arthritis (versus no)**	-0.43 [-0.52, -0.34]	0.96 [0.87, 1.05]	-0.47 [-0.73, -0.21]	0.77 [0.59, 1.01]
**Ankylosing spondylitis (versus no)**	0.16 [0.09, 0.24]	0.99 [0.91, 1.07]	0.24 [0.05, 0.44]	0.98 [0.79, 1.20]
**Neurocardiovascular diseases (versus no)**	-0.42 [-0.45, -0.40]	0.95 [0.92, 0.97]	-0.28 [-0.35, -0.22]	0.93 [0.86, 1.01]
**Diabetes (versus no)**	-0.20 [-0.22, -0.18]	1.10 [1.07, 1.14]	-0.12 [-0.19, -0.05]	1.10 [1.02, 1.19]
**Obesity (versus no)**	0.07 [0.05, 0.10]	1.11 [1.08, 1.15]	0.22 [0.15, 0.30]	1.14 [1.06, 1.24]
**Active cancers (versus no)**	0.13 [0.10, 0.16]	1.32 [1.27, 1.36]	0.45 [0.37, 0.53]	1.55 [1.42, 1.70]
**Disc disease (versus no)**	0.05 [-0.01, 0.11]	0.72 [0.67, 0.76]	0.07 [-0.11, 0.25]	0.74 [0.61, 0.89]
**Carpal tunnel syndrome (versus no)**	-0.17 [-0.26, -0.08]	0.88 [0.80, 0.97]	-0.10 [-0.37, 0.16]	0.93 [0.71, 1.22]
**Nerve root compression (versus no)**	0.01 [-0.08, 0.10]	0.81 [0.74, 0.89]	-0.26 [-0.58, 0.06]	0.60 [0.43, 0.84]

**Chronic end-stage renal disease (versus no)**	-1.83 [-2.06, -1.60]	0.20 [0.16, 0.25]	-1.97 [-2.71, -1.23]	0.13 [0.06, 0.29]
**Spinal fracture (versus no)**	0.01 [-0.12, 0.14]	1.00 [0.87, 1.15]	0.16 [-0.22, 0.54]	1.22 [0.81, 1.85]
**Migraine (versus no)**	0.26 [0.10, 0.42]	0.81 [0.68, 0.96]	0.50 [0.13, 0.86]	1.10 [0.75, 1.61]
**Prior exposure to pregabalin (versus no)**	NA	NA	0.55 [0.49, 0.60]	1.52 [1.43, 1.61]
**Prior exposure to gabapentin (versus no)**	0.66 [0.62, 0.69]	1.92 [1.85, 1.99]	NA	NA
**Prior exposure to weak opioid analgesics (versus no)**	0.09 [0.07, 0.11]	1.00 [0.98, 1.02]	0.21 [0.15, 0.27]	1.04 [0.98, 1.11]
**Prior exposure to strong opioid analgesics (versus no)**	0.34 [0.31, 0.36]	1.27 [1.24, 1.31]	0.61 [0.55, 0.68]	1.44 [1.34, 1.55]
**Prior exposure to antiepileptics (versus no)**	0.49 [0.45, 0.52]	1.19 [1.14, 1.25]	0.47 [0.38, 0.55]	1.27 [1.15, 1.41]
**Prior exposure to antipsychotics (versus no)**	0.66 [0.63, 0.69]	1.19 [1.15, 1.24]	0.25 [0.15, 0.36]	0.97 [0.86, 1.10]
**Prior exposure to anxiolytics (versus no)**	0.22 [0.20, 0.24]	1.06 [1.03, 1.08]	0.13 [0.07, 0.18]	0.94 [0.88, 1.00]
**Prior exposure to hypnotics and sedatives (versus no)**	0.25 [0.22, 0.27]	1.19 [1.16, 1.22]	0.22 [0.15, 0.28]	1.09 [1.02, 1.18]
**Prior exposure to antidepressants (versus no)**	0.10 [0.08, 0.12]	0.94 [0.92, 0.96]	0.32 [0.26, 0.37]	1.11 [1.04, 1.18]
**Prior exposure to opioid agonist treatments (versus no)**	2.30 [2.26, 2.34]	3.30 [3.12, 3.48]	1.02 [0.77, 1.28]	1.42 [1.09, 1.86]

Supplementary Figure 1. Cumulative incidence of high-dose use of pregabalin and gabapentin. a) with a 42-day threshold to define treatment interruptions (log-rank test: p < 0·001). b) with a 56-day threshold to define treatment interruptions (log-rank test: p < 0·001). We defined time-to-event as the time from cohort entry to the midpoint of the first treatment episode where high-dose use was identified. Therefore, the bump at 12 months corresponds to the time-to-event for patients with a single 24-month treatment episode. With longer thresholds, more patients have a single 24-month treatment episode, resulting in a more marked bump.

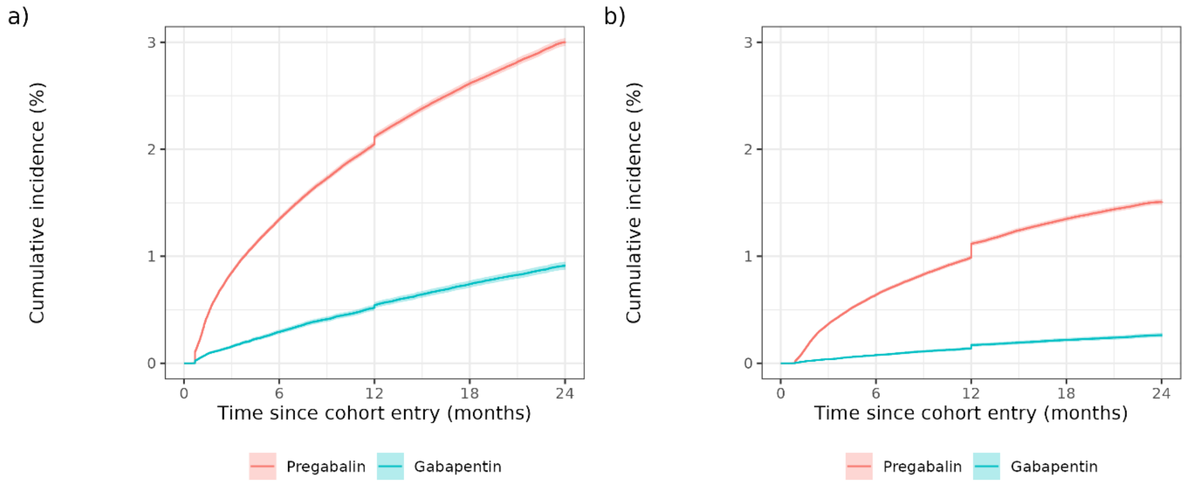

Number at risk (censored) — a)

	0	6	12	18	24
Pregabalin	898,887 (0)	865,696 (21,176)	833,559 (47,371)	807,551 (68,477)	785,960 (535,960)
Gabapentin	271,832 (0)	265,876 (5,167)	258,507 (11,979)	251,980 (17,898)	246,232 (181,420)

Number at risk (censored) — b)

	0	6	12	18	24
Pregabalin	898,887 (0)	871,847 (21,343)	842,500 (47,807)	817,924 (69,193)	797,952 (543,517)
Gabapentin	271,832 (0)	266,433 (5,194)	259,460 (12,042)	253,265 (17,991)	247,806 (182,542)

Supplementary Figure 2. Estimated time-dependent hazard ratios and time-averaged hazard ratios for two-levels covariates associated with high-dose use of pregabalin. The dotted lines correspond to a null effect.

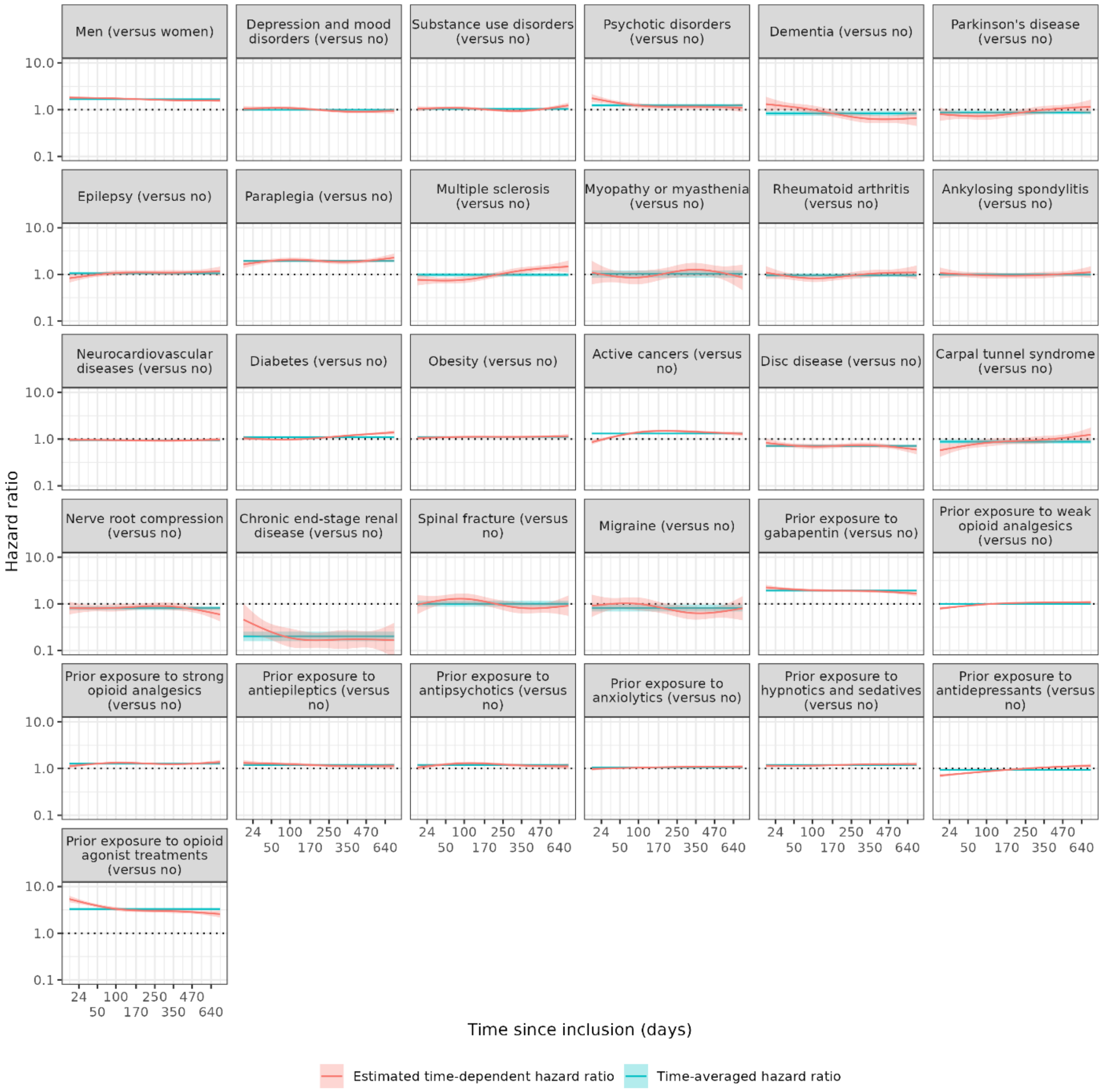

Supplementary Figure 3. Estimated time-dependent hazard ratios and time-averaged hazard ratios for two-levels covariates associated with high-dose use of gabapentin. The dotted lines correspond to a null effect.

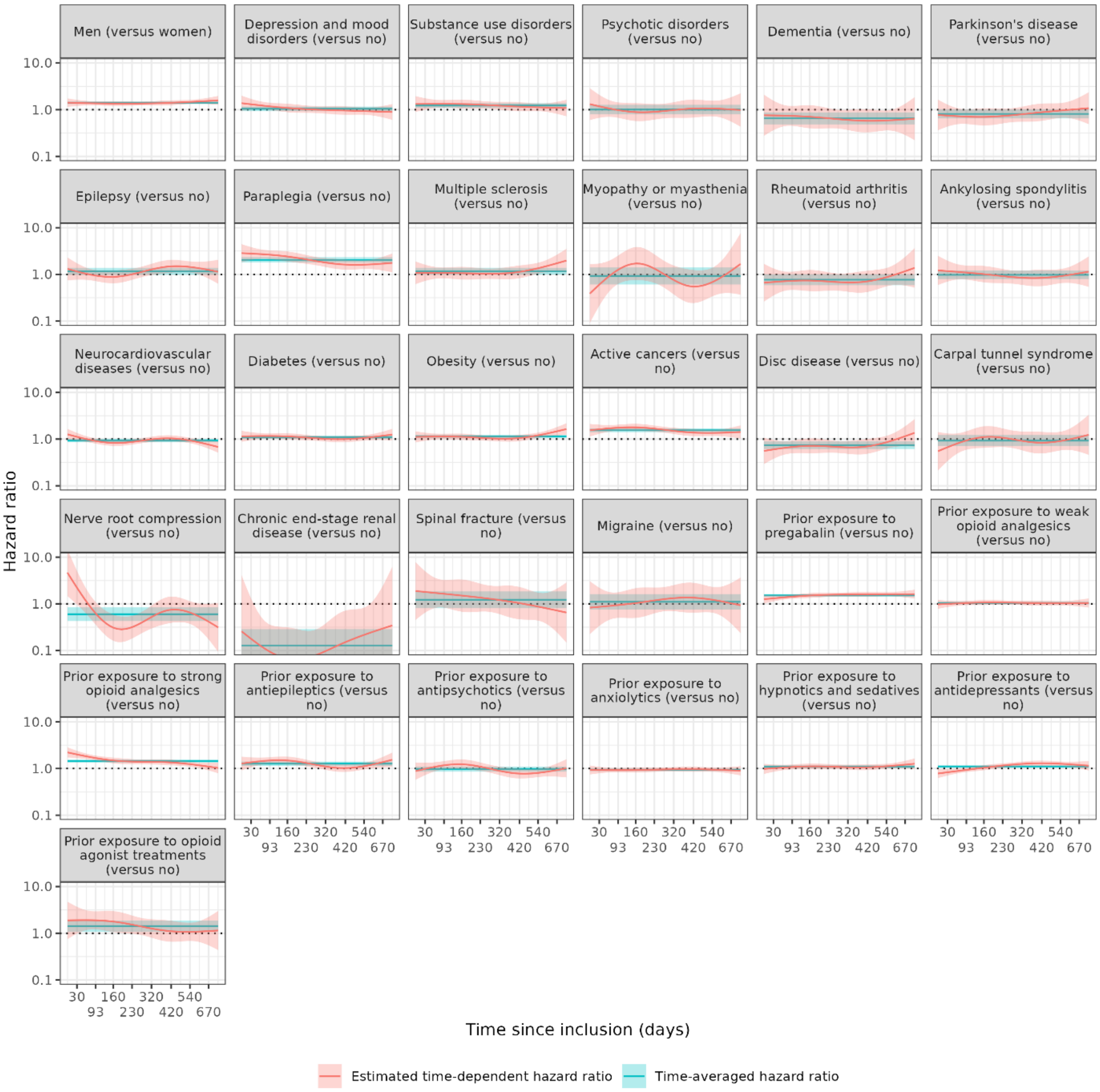

Supplement: Supplementary Figs. S1–S3 and Tables S1–S3 [file mmc1.pdf]
